# Metabolic reprogramming and crosstalk of cancer-related fibroblasts and immune cells in the tumor microenvironment

**DOI:** 10.3389/fendo.2022.988295

**Published:** 2022-08-15

**Authors:** Yifei Zhu, Xinyan Li, Lei Wang, Xiwei Hong, Jie Yang

**Affiliations:** ^1^ School of Medicine, Southeast University, Nanjing, China; ^2^ Department of General surgery, Affiliated Kunshan Hospital of Jiangsu University, Kunshan, China

**Keywords:** Tumor microenvironment, cancer-associated fibroblasts, immune cells, metabolic reprogramming, immunotherapy

## Abstract

It is notorious that cancer cells alter their metabolism to adjust to harsh environments of hypoxia and nutritional starvation. Metabolic reprogramming most often occurs in the tumor microenvironment (TME). TME is defined as the cellular environment in which the tumor resides. This includes surrounding blood vessels, fibroblasts, immune cells, signaling molecules and the extracellular matrix (ECM). It is increasingly recognized that cancer cells, fibroblasts and immune cells within TME can regulate tumor progression through metabolic reprogramming. As the most significant proportion of cells among all the stromal cells that constitute TME, cancer-associated fibroblasts (CAFs) are closely associated with tumorigenesis and progression. Multitudinous studies have shown that CAFs participate in and promote tumor metabolic reprogramming and exert regulatory effects *via* the dysregulation of metabolic pathways. Previous studies have demonstrated that curbing the substance exchange between CAFs and tumor cells can dramatically restrain tumor growth. Emerging studies suggest that CAFs within the TME have emerged as important determinants of metabolic reprogramming. Metabolic reprogramming also occurs in the metabolic pattern of immune cells. In the meanwhile, immune cell phenotype and functions are metabolically regulated. Notably, immune cell functions influenced by metabolic programs may ultimately lead to alterations in tumor immunity. Despite the fact that multiple previous researches have been devoted to studying the interplays between different cells in the tumor microenvironment, the complicated relationship between CAFs and immune cells and implications of metabolic reprogramming remains unknown and requires further investigation. In this review, we discuss our current comprehension of metabolic reprogramming of CAFs and immune cells (mainly glucose, amino acid, and lipid metabolism) and crosstalk between them that induces immune responses, and we also highlight their contributions to tumorigenesis and progression. Furthermore, we underscore potential therapeutic opportunities arising from metabolism dysregulation and metabolic crosstalk, focusing on strategies targeting CAFs and immune cell metabolic crosstalk in cancer immunotherapy.

## Introduction

The term “metabolic reprogramming” is often used to denote a set of abnormal metabolic pathways observed in highly proliferative tumor or cancer cells (1). In cancer, malignant cells acquire metabolic adaptations through various extracellular and endocytic pathways to meet the necessary nutrients required for tumor growth and to use these nutrients to maintain survival and produce new biomass (2, 3). Some of these adaptations initiate the transformation process, and some promote the growth of malignant cells, making them vulnerable to inhibitors of key pathways (4). Cancer cells depend on a variety of different metabolic pathways and the specific nutrients used are influenced by both cancer cell genes and environmental conditions (5). The vast majority of mammalian cells use glucose as a source of energy. Glucose is metabolized by glycolysis, which undergoes multiple reaction steps to yield pyruvate. In typical cells with normal blood oxygen levels, most of the pyruvate gets access in the mitochondria. It is oxidized by the tricarboxylic acid cycle (TCA) to generate ATP to satisfy the cell’s energy needs. However, in highly proliferative cell types such as cancer cells, the vast majority of the pyruvate produced by glycolysis leaves the mitochondria. It produces lactate by lactate dehydrogenase (LDH/LDHA), a procedure that usually occurs in the hypoxic state. The product of lactic acid in the existence of oxygen is called “aerobic glycolysis” or “the Warburg effect” (2, 3, 6–11).

Tumor microenvironment (TME) is identified as the surrounding microenvironment where tumor cells reside, including surrounding blood vessels, fibroblasts, immune cells, bone marrow-derived inflammatory cells, various signaling molecules and ECM. TME is a complicated integrated system made up of the metabolic reprogramming of tumor cells with surrounding tissues and immune cells, as well as the crosstalk between them. It has long been identified that TME acts as a pivotal position in the development and progression of tumors. Although other reviews have provided a detailed examination of the role of the TME in tumorigenesis (12–15), here, we concentrate on the metabolic reprogramming in cancer-associated fibroblasts (CAFs) and immune cells along with the crosstalk between them that induces immune responses, and we also highlight the therapeutic opportunities presented by metabolic dysregulation and metabolic crosstalk, focusing on strategies that may help to precisely target alterations in tumor metabolism.

Fibroblasts play a prominent role in tissue homeostasis, tumorigenesis, and inflammatory and fibrosis progression (16). CAFs are one of the most plentiful matrix components in TME and become specific targets in the vast majority of solid tumors (17). A large number of researches have illustrated that CAFs play protruding roles in tumor pathogenesis and have important clinical significance (16, 18–21). Mechanistically and functionally, CAFs secrete various cytokines or metabolites through metabolic reprogramming to inhibit the function of immune cells and promote tumor development, invasion, and metastasis; CAFs also have the ability to shape the EMC, form a barrier for drug or therapeutic immune cell penetration and prevent the deep penetration of drugs and immune cells into tumor tissues, thus reducing the effectiveness of tumor treatment (22–25). In cancer, a greater understanding of the complexity of CAFs may have therapeutic and prognostic value. Various types of adaptive and innate immune cells exist or infiltrate into TME. The metabolic reprogramming between these immune cells and tumor cells determines the immune status of the tumor and can promote or suppress the immune response of the tumor (26). To better understand the role of this metabolic crosstalk in TME, this review discusses the metabolic reprogramming of CAFs and immune cells, as well as their interactions and further highlights the meaning of developing novel therapeutic strategies on the basis of metabolism.

## Cancer-associated fibroblasts sources, heterogeneity and metabolic reprogramming

### Cellular origin of CAFs: known & unknown

Previous studies have demonstrated that CAFs activation and reprogramming can occur in TME (17, 27–29). Therefore, identifying the cellular origin of CAF subtypes is a critical issue, as it may partly confirm the functions among different CAF populations and may enlighten new therapeutic strategies.

**Figure 1:**
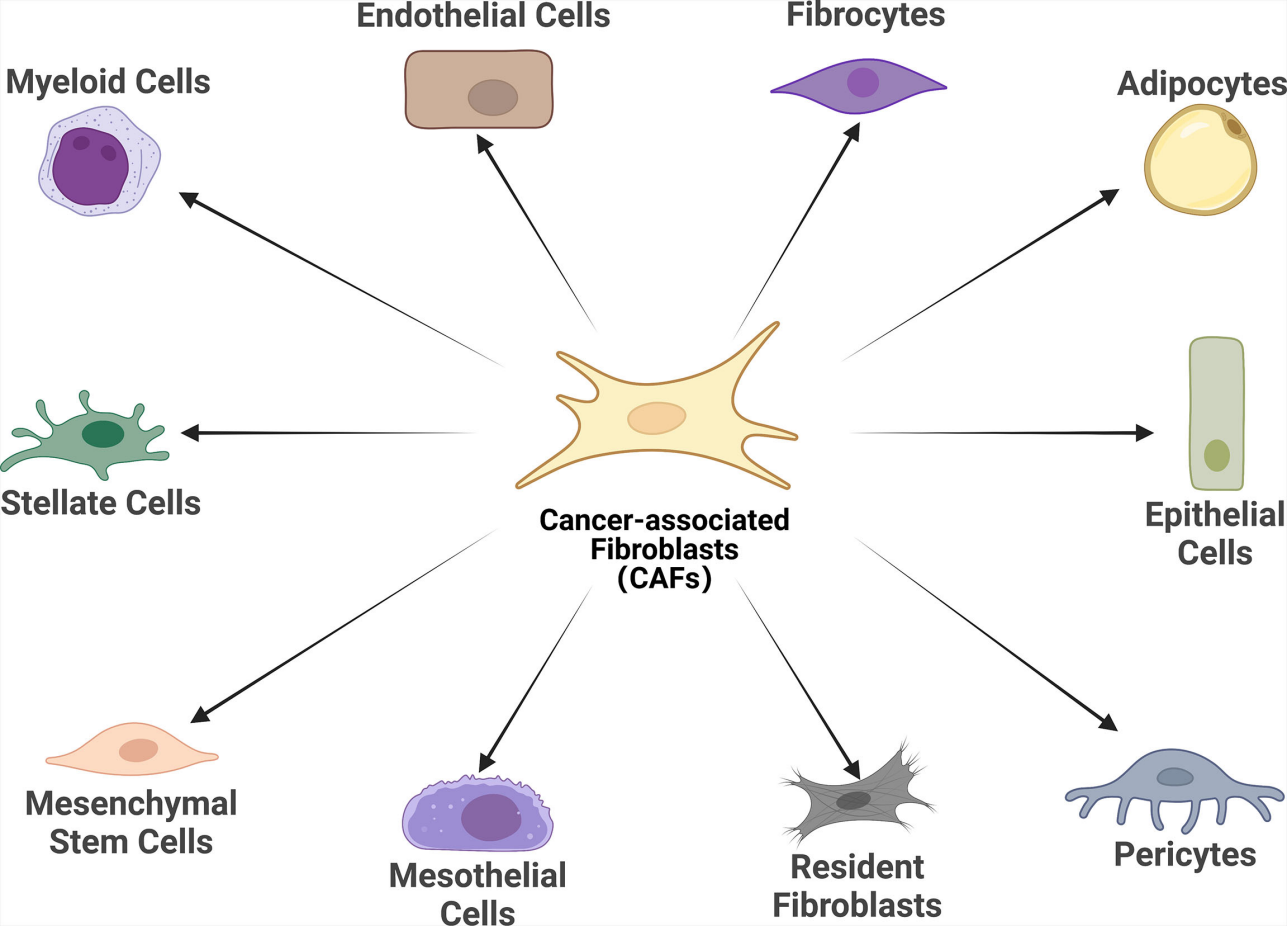
Cell of origin of cancer-associated fibroblasts (CAFs). Schematic representation of the cells of origin of CAFs that have been reported or in potential, including epithelial cells, mesothelial cells, resident fibroblasts, stellate cells, pericytes, adipocytes, mesenchymal stem cells, myeloid cells, fibrocytes, and endothelial cells.

There is growing evidence that CAFs is a complex heterogeneous cell population, which may be attributed to the diverse potential cellular origins of CAFs (30, 31) (**Figure 1**). Resting tissue fibroblasts can form CAFs upon activation by neighboring tumor cells. For instance, resting hepatic stellate cells (HSCs) and pancreatic stellate cells (PSCs) are separately considered as CAFs in pancreatic and hepatocellular carcinomas (32–35). Some other major cell sources of CAFs identified in different studies include mesenchymal stem cells, endothelial cells and adipocytes (36–44). Notably, although CAFs and tumor-associated mesenchymal stem cells (TA-MSCs) are derived from the same cells (primitive mesenchymal stem cells), TA- MSCs have greater self-renewal capability and low expression of markers associated with CAFs such as wave proteins, fibroblast activation protein (FAP) and fibroblast-specific proteins 1 (FSP1, or called S100A4), which warrants further mechanistic studies (45–47). Overall, the exact origin of CAFs and their subpopulations have not been fully elucidated. Probably because of these cells’ phenotypic and functional plasticity and the lack of specific genealogical biomarkers. The development of genealogical tracing techniques may help better to trace the origin of CAFs in the coming years.

Generally speaking, CAFs are considered to be genetically stable, especially when it comes to tumor cells (48, 49). Still, certain DNA damage, such as radiation, can lead to the transformation of normal fibroblasts to CAFs, which is reminiscent of tumorigenic development (50). Future studies on the role of genetic mutations in CAFs need to be more in-depth. However, to date, the origin of some CAFs, the cancer-restraining subgroups (rCAFs) in particular, remains unclear, and further exploration of rCAFs may turn into a prospective research direction.

### Heterogeneity of CAFs in malignancies

The heterogeneity of CAFs, especially those activated from the resting state, may depend on their origin. Because of their different origins, the functions of these activated fibroblasts may be diverse (19, 30). CAFs are highly plastic and pluripotent. Activated CAFs can adapt to perivascular and vascular functions. This plasticity that leads to the heterogeneity of CAFs (19). The observed heterogeneity and plasticity of CAFs may have the following possible explanations (1): dynamic and interchangeable changes in CAFs between tumor-promoting or tumor-suppressing phenotypes, counting on the intricate background of the surrounding TME (2); the existence of a wide range of CAF subpopulations with different functions, and the diversity of CAF subpopulations may exceed the few major subpopulations defined in previous studies (51). Many MSC biomarkers can selectively identify activated CAFs in specific tumor microenvironments (28).

Over the past few years, it has been possible to identify a variety of biomarker genes that define different potential subgroups of CAFs with the advent of single-cell RNA sequencing (scRNA-seq) (52), leading to a deeper knowledge of the plasticity and heterogeneity of CAFs exhibited in various tumor types. Different biomarkers and classification approaches are often used to classify tumor types and various CAF subgroups. Here, we summarize common tumors and the corresponding CAF subgroups according to the latest studies (**Table 1**) (21, 53–74). The expression of surface markers varies among different subtypes of CAFs. For example, FAP is considered to be a CAFs-specific expressed protein, and therefore FAP is often used as a target of CAFs for tumor diagnosis and treatment (53, 75, 76). α-SMA has serine protease activity and can be involved in fibrillogenesis and ECM remodelling, thus enhancing the malignant behavior of activated tumor cells. α- can be found in most cancer types. α-SMA not only assists in recognizing activated CAFs, but also assumes the role of a universal marker for some mesenchymal cells. Therefore, α-SMA is usually used as a major evaluation criterion for the prognosis of targeted CAFs treatment (30, 77, 78). Another renowned marker is fibroblast-specific protein, which, as the name suggests, is expressed comparatively specifically on fibroblasts. CAFs with the expression of FSP1 have a unique tumor-protective role in immunosurveillance because such CAFs can produce collagen (79–81).

**Table 1:** The proposed classification of CAFs in different cancer types.

Cancer type	Reference	CAF subpopulation	Biomarkers
Breast cancer	Bartoschek^52^	Vascular CAFs (vCAF)	DES, Nidogen-2
		Matrix CAFs (mCAF)	Fibulin-1, PDGFRα, CXCL14
		Cycling CAFs (cCAF)	Ki-67,
		Developmental CAFs (dCAF)	SCRG1, PyMT
	Brechbuhl^53^	CD146^+^CAF	thrombospondin 1, COL18A1
		CD146^-^CAF	FN1, TNC
	Costa^54^, Givel^55^, Pelon^56^	CAF-S1	FAP, S100-A4/FSP1
		CAF-S2	NA
		CAF-S3	PDGFRβ, FSP1
		CAF-S4	CD29
	Wu^57^	Inflammatory CAFs (iCAF)	CXCL12
		Myofibroblast CAFs (myCAF)	ACTA2, FAP, PDPN, COL1A1, COL1A2
	Friedman^58^	PDPN-CAF	CXCL12, SAA3, CXCL1, IL-6
		S100A4-CAF	HSPD1, SPP1
Lung cancer	Lambrechts^59^	Cluster-1	NA
		Cluster-2 (myofibroblast)	α-SMA
		Cluster-4	NA
		Cluster-5	NA
		Cluster-7	NA
	Hao^60^	HD-CAF	NA
		LD-CAF	NA
	Su^61^	CD10+GPR77+CAF	α-SMA, FAP
Pancreatic cancer	Öhlund^30^	myCAF	α-SMA, FAP
		iCAF	IL-6
	Ligorio^62^	EMT-CAF	Ki67
		PRO-CAF	FN1
	Elyada^63^	Antigen-presenting CAFs (apCAF)	CD47, MHC
	Bernard^64^, Hosein^65^, Peng^66^	myCAF	α-SMA, THY1, CTGF
		iCAF	COL14A1, LY6C, CLEC3B
		apCAF	CD74, SAA3, FSP1
Colorectal cancer	Li^67^, Zhang^68^	CAF-A (FAP-CAF)	FAP, DCN, MMP-2
		CAF-B (α-SMA-CAF)	α-SMA, TAGLN, PDGFA
Melanoma	Davidson^69^	CAF-S1	Immune CAHs, CD34
		CAF-S2	Desmoplastic CAFs, TNC
		CAF-S3	Contractile CAFs, α-SMA
Prostate cancer	Chen^70^	CAF-S1	α-SMA, PDGFRβ
		CAF-S2	PDGFRα, PLAGL1, CREB3L1
		CAF-S3	α-SMA, MAFB, HOXB2
Head and neck squamous cell carcinoma (HNSCC)	Puram^71^	CAF1	NA
		CAF2	NA
Bladder cancer	Chen^72^	myo-CAF	RGS5, MYL9, MYH11
		iCAF	PDGFRα, CREB3L1, PLAGL1
Cholangiocarcinoma^54^	Affo^73^	myCAF	COL1A1, α-SMA, COL8A1
		mesCAF	Mesothelin
		iCAF	CXCL12, HGF, RGS5
Gastric cancer	Li^74^	iCAF	IL-6, CXCL12
		Extracellular matrix CAFs (eCAF)	POSTN

CAF, cancer-associated fibroblasts; CAV1, Caveolin-1; ECM, extracellular matrix; EMT, epithelial-mesenchymal transition; FAP, fibroblast activation protein; FSP-1, fibroblast specific protein 1; CXCL2 C-X-C chemokine ligand 2; CCL2 C–C chemokine ligand 2; MHC class II major histocompatibility complex class II; POSTN periostin; PDPN, podoplanin; PDGFR, platelet-derived growth factor receptor; α-SMA, α-smooth muscle actin; COL1A2 collagen type 1 Alpha 2; PDGFA platelet derived growth factor A; PDGFRβ, platelet- derived growth factor receptor-β; CXCR4, CXC- chemokine receptor 4.

Similar to the mode of operation of T lymphocytes, CAFs consist of many cell subsets that respond to different metabolic and immune responses, form specific secretory manifestations, and perform irreplaceable biological functions in TME. Although CAF markers are diverse, identifying the functional subset of CAFs with the application of cell surface markers remains arduous. The development of *in vitro* CAF functional and mechanistic studies of live cell sorting together with *in vivo* CAF-targeted therapies have been significantly hampered by the absence of well-defined cell surface markers. It is undeniable that single-cell detection technology has made significant advancements in the past years, the variety of markers for CAFs is abundant, and the genetic signature of specific CAF subtypes may vary between cancer types, and different stages of certain types of cancer, or between patients. These pose a great challenge to differentiate CAF subtypes and identify specific and accurate cell surface markers.

### Metabolic reprogramming of CAFs

**Figure 2:**
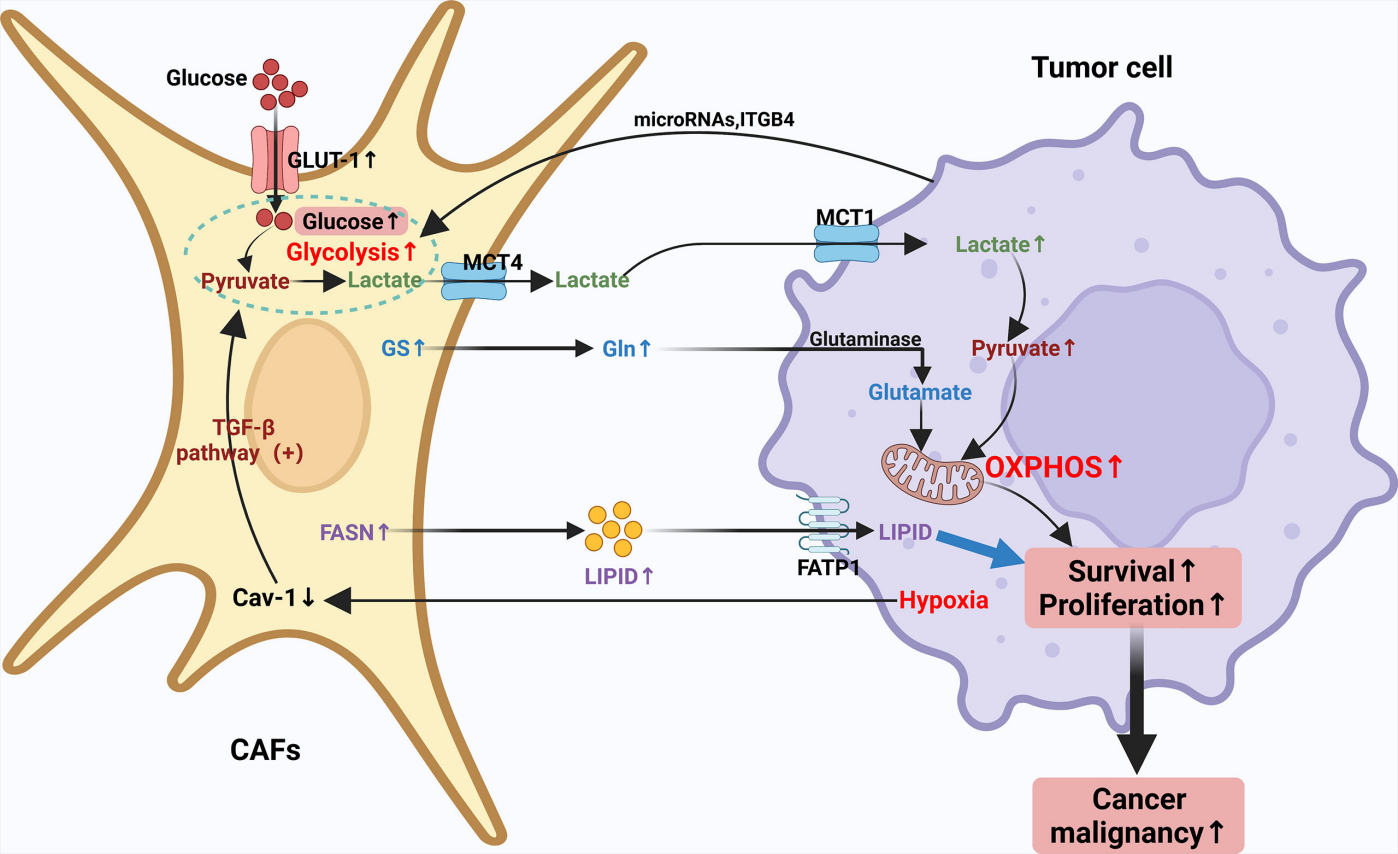
Crosstalk of metabolic reprogramming between CAFs and tumor cell. Both cancer cells and CAFs undergo a regulated metabolic reprogramming in the tumor microenvironment, in particular, CAFs undergo a clear Warburg effect. Typically, tumor cells increase their uptake of various nutrients and enhance their metabolic rate, with glucose and glutamine being the most prominent components. CAFs have been shown to be involved in the complex metabolism of tumors, mainly including glucose, amino acid and lipid metabolism, prompting tumor cells to counteract energy depletion due to the Warburg effect. The regulation of CAFs through these metabolic switches forms a unique CAF behavior and leads to altered tumor cell behavior through the unique regulation of CAFs through these metabolic switches.

In recent years, it has been increasingly recognized that malignancies can be considered not only genetic diseases but also metabolic diseases (82, 83). Metabolic reprogramming meets the high demand of tumor cells for rapid proliferation and growth and assists tumor cells in surviving in the relatively low oxygen and hostile environment (83). Malignant cells require sufficient Adenosine Triphosphate (ATP), as well as several other nutrients, including nucleic acids, lipids and proteins to sustain their growth. Previous studies have found that cancer cells tend to meet the need for additional energy through metabolic reprogramming as a way to maintain a sustained state of cell proliferation and hypo differentiation (84). As the most profoundly understood cancer metabolic modality or pathway to date, the Warburg effect, meticulously depicts the metabolic reprogramming of cancer cells. In a specific TME setting, CAFs may be involved in the metabolic balance of synthesis and catabolism in conditional cancer cells (84–87). The cellular metabolism of CAFs is very similar to that of continuously proliferating cells and both are dependent on aerobic glycolysis (7). The Warburg effect is more pronounced in CAFs and seems to be associated with increased cellular catabolic activity and cellular autophagy. The interactions between cancer cells and CAFs are inextricably linked to metabolic reprogramming, which promotes the growth of cancer cells, metastasis and absconds of immune surveillance (88). However, this interaction’s detailed mechanisms and specific processes of this interaction are still unclear. Typically, tumor cells increase the uptake and the metabolic rate of various nutrients, of which glucose and glutamine are the most prominent components. CAFs have been shown to be involved in the elaborate metabolism of tumors, mainly containing glucose, amino acid and lipid metabolism, prompting tumor cells to counteract energy depletion due to the Warburg effect. The regulation of CAFs through these metabolic switches shapes the unique code of CAFs through these metabolic switches shapes the unusual CAF behavior and leads to altered tumor cell behavior (18, 31, 89–91). Therefore, an in-depth study of the metabolic reprogramming of CAFs can help to understand tumor cell growth and metastasis better. The metabolic reprogramming of CAFs will be discussed in the following aspects (**Figure 2**).

#### Glucose metabolism

CAFs have shifted to a more glycolysis-dependent phenotype through metabolic reprogramming, whereas normal cells count more on the mitochondrial pathway to generate energy *via* mitochondrial oxidative phosphorylation (OXPHOS). Moreover, it has been demonstrated that repression of glycolysis in tumor cells can recover OXPHOS (92–95). Under certain circumstances, energy supply through mitochondrial respiration is responsible for 80% of the ATP required by certain types of cancer cells such as MCF7 breast cancer (2, 96–99). However, the specific mechanism remains unclear currently, one possible explanation is that tumor cells that rely primarily on OXPHOS metabolism for energy supply, achieve resistance to oxidative stress by enhancing antioxidant responses and increasing detoxification capacity (100–102). Some of the enzymes involved in the glycolytic process, for example, HK2 and 6-phosphofructokinase liver type (PFKL) are considerably up-regulated in CAFs, which corroborates their glycolytic properties. HK2, as a fundamental glycolytic enzyme, is overexpressed in tumors, resulting in the “Warburg effect”. In the CAF model, the level of HK2 protein is apparently up-regulated during the differentiation of CAFs (103–106).

In the past, the high glycolytic rate of CAFs was often considered to be one of the main drivers for the maintenance of uninterrupted growth and proliferation of tumor cells. However, the specific biomolecular mechanisms underlying the occurrence of such high glycolytic rates in CAFs have not been fully understood to date. Encouragingly, lots of potential mechanisms have been put forward and further investigated to illustrate the metabolic reprogramming correlated with the upregulation of glycolysis in CAFs (107). One of the more accepted theories is that during contact with cancer cells, CAFs are reprogrammed to develop a glycolysis-dependent phenotype that increases glucose uptake and utilization and facilitates the transport of pyruvate and lactate (end products of glycolysis) (106). Lactate is expeditiously and effectively utilized by the cancer cells to gain energy and molecules through strengthened anabolism and to fuel OXPHOS (108). The above evidence mentioned offers further perception into the conception of metabolic reprogramming in TME.

Glucose enters cells through upregulation of glucose transporter 1 (GLUT-1) expression. Past studies have found that oncogenes like cMyc enhance glucose uptake by increasing lactate dehydrogenase a (LDHA) and GLUT-1 expression as a way to increase metabolic flux (109). It has also been documented that the promoters of pyruvate kinase (PK) and lactate dehydrogenase (LDHA) genes, key enzymes in the glycolytic process, are highly methylated in breast cancer-associated CAFs, which increases pyruvate kinase M2 (PKM2) and LDHA expression (105). Furthermore, changes in miR-186 expression during the formation of CAFs result in altered protein levels of GLUT-1. Thus, miR-186 can regulate glycolysis through changes in GLUT-1 expression (110). Decreased expression of isocitrate dehydrogenase 3 (IDH3α) α subunit has been reported to be connected with the metabolic transition from OXPHOS to glycolysis, and IDH3α overexpression prevents fibroblasts from converting to CAFs (105). Downregulation of Caveolin-1 (Cav-1) may give rise to the activation of the TGF-β pathway in CAFs, and the activated pathway allows for enhanced oxidative stress and aerobic glycolytic responses. Cav-1-deficient CAFs facilitate tumor cell development and aberrant vascular branching. Proteomic analysis of Cav-1-deficient CAFs demonstrates upregulation of glycolytic enzymes (e.g. PKM2 and LDH-B) (111–113). The investigators found that Cav-1 dysregulation brings about aberrant mitochondrial transcription factor A (TFAM) expression in fibroblasts, which in turn induces oxidative stress, mitochondrial dysfunction and aerobic glycolysis in TME (103). CAFs lacking TFAM may yield more hydrogen peroxide and L-lactic acid by paracrine means providing energy-rich metabolites for the purpose of promoting tumor growth and angiogenesis (114). Finally, it has been found that fibroblasts knocking down Cav-1 may exert an influence on cancer cell metabolism by promoting the production of lactate, a mitochondrial respiration product of synthetic cancer cells (103, 115). Overall, Cav-1 deficiency is critical in the metabolic reprogramming of CAFs. Some other markers such as integrin- 4 (ITGB4) in triple-negative breast cancer (TNBC) (113), MCT4 in nasopharyngeal carcinoma (116), MCT1 and MCT4 in breast and bladder cancer (117, 118), ITGB2 in oral squamous carcinoma (OSCC) (119), MCT1, succinate dehydrogenase (SDH) and fumarate hydratase (FH) expression levels are significantly elevated in pancreatic cancer cells (120). The overexpression of these markers further suggests metabolic crosstalk between CAFs and cancer cells.

#### Amino acid metabolism

Besides glucose, previous studies have proven that CAFs can raise the output of other nutrients required by cancer cells, such as amino acids that may function as a source of anabolic or fuel for OXPHOS, as cancer cells need more amino acids to meet their demand for rapid proliferation (121–124). Multiple studies have demonstrated that CAFs compound certain amino acids through the TCA cycle to maintain the continuous proliferation of tumor cells (125, 126). Amino acids can be separated into two classifications: essential amino acids and non-essential amino acids. Amino Acids have been widely studied for their use in the synthesis of proteins, peptides and other nitrogenous substances that are unique to the body, as well as for their role as metabolites that regulate the rapid and uninterrupted proliferation of cancer cells, with glutamine being the most studied (127, 128). Glutamine (Gln), an amide of glutamate, is one of the non-essential amino acids (which can be produced by the body without relying on diet), a major source of carbon and nitrogen, and has a role in promoting almost every biosynthetic pathway in cancer cells (122).

Previous studies have shown that exosomes derived from patients with prostate and pancreatic cancer can inhibit mitochondrial OXPHOS and compensate for increased glycolysis (129). Furthermore, it was demonstrated that exosomes are a source of metabolites carrying lactate, amino acids, TCA cycle intermediates and lipids that cancer cells utilize to proliferate and replenish levels of TCA cycle metabolites (130). Researchers found that these exosome-fed prostate cancer cells also presented a greater reliance on glutamine, with significantly raised levels of ^13^C-labeled m + 5 glutamate and m + 5 α-KG through ^13^C5-glutamine labeling experiments. This outcome demonstrates that CAFs have the ability to shift the metabolic pattern of cancer cells from mitochondria-reliable to glycolysis-reliable and up-regulate glutamine metabolism as a means of feeding the proliferating cancer cells with nutrients (129, 131, 132). In other cancers, such as ovarian cancer, CAFs produce Gln in large amounts *via* glutamine synthetase (GS). the Gln produced through the metabolism of CAFs is delivered to ovarian cancer cells, where Gln provides a source of nutrients for cancer cells on the one hand, and is converted to glutamate *via* glutaminase on the other hand, further supporting tumor cell growth by supplementing TCA metabolic pathway intermediates (133). Glutamine addiction is the process by which glutamine enters the TCA cycle to provide energy and growth for proliferating cancer cells (124). In recent studies, glutamine dependence was found to drive CAFs to migrate from nutrient-depleted regions where glutamine is about to be depleted to more glutamine-rich regions (134, 135). In a way, glutamine dependence accelerates the migration and aggression of CAFs, which boosts the migration of cancer cells to the eutrophic area in turn. The transfer of CAFs to Gln-rich areas is mediated by a polarized protein kinase B (AKT2), and the presence of polarized AKT2 recruits the aggression of CAFs and the absconds of cancer cells from original tumor sites (134). However, more studies are needed to demonstrate whether AKT2 can be a target for metabolic reprogramming therapy targeting CAFs. The specific roles of numerous amino acids in CAF metabolic reprogramming have yet to be determined. Further research is needed to paraphrase the metabolic roles of other amino acids in cancer cell growth and proliferation. These findings open up opportunities for metabolic reprogramming therapies targeting CAFs.

#### Lipid metabolism

Lipids are defined as natural compounds that are soluble in non-polar solvents but insoluble in water (136). The most important components of lipids are fatty acids (FAs), which are not merely essential composition of cell membranes but also serve as important precursors of second messengers involved in transducing intracellular signals and as an important source of energy when the main energy donor is restricted (137–140). Few previous studies have investigated the effects of abnormal lipid metabolism of CAFs on tumor growth and metastasis. Recent studies indicate that CAFs may play a fundamental role in lipid translocation and uptake. It has been documented that CAFs induce FATP1 upregulation in human MDA-MB-231 TNBC cells, leading to increased absorption of exogenous fatty acids by TME (141). CAFs can also transport lipids into cancer cells *via* exosomes, which have been proven to boost cancer cell growth and proliferation (142). Previous studies have shown that CAFs can undergo reprogramming of lipidome and cumulate more fatty acids and phospholipids, thereby promoting colorectal cancer (CRC) cell migration. CAFs increase CRC cell migration even after protein deprivation, suggesting that non-protein molecular metabolites in CAFs are accountable for CRC cell migration, and these non-protein molecular metabolites may be CAFs metabolic reprogramming. These non-protein metabolites may be lipid metabolites secreted by CAFs after metabolic reprogramming (142–144). Fatty acid synthase (FASN), one of the essential enzymes in fatty acid composite, is dramatically increased in CAFs, thus increasing the production of FAs (82). Recent studies have confirmed that the CRC cell-derived exosome HSPC111 promotes CRC cell migration by reprogramming lipidomic metabolism in CAFs, suggesting that HSPC111 has the tendency to be a potential therapeutic target for the prevention of CRC cell metastasis (144, 145).

## Immune cells in the tumor immune microenvironment

In recent years, numerous studies have demonstrated that TME plays an essential role in tumor immunosuppression, targeted therapy and other responses, and has gained widespread attention (146–148). The presence of different immune cell populations in TME, including various innate immune cells (macrophages, dendritic cells, innate lymphocytes and NK cells, etc.) and adaptive immune cells (T cells and B cells, etc.), is highly relevant to the anti-tumor immune status is highly correlated (76, 149, 150). When TME is affiliated with the function and signaling of these immune cells, it is also referred to as the tumor immune microenvironment (TIME) (151), which controls the development, evolution and metastasis of the tumor (149, 152, 153). Therefore, the use of good immune cells is of great importance in the treatment of tumors. Here, we list the functions and metabolic phenotypes of several common immune cells (**Table 2**).

**Table 2:** Functions and metabolic phenotypes of immune cells.

Immune cell type	Subtypes	Function	Metabolic patterns
T cell	Naïve T cell	Mature in the thymus and migrate to peripheral lymphoid tissue, identify antigens and differentiate into Teff	OXPHOS FAO Glutamine metabolism
	Treg cell	Anti-Teff to maintain immune tolerance and prevent the occurrence of autoimmune diseases	OXPHOS FAO
	Memory T cell	Protect against reinfection or tumor re-emergence	FAO OXPHOS
	Effector T cell	Secrete lymphokines and perform cellular immunity	Glycolysis OXPHOS
	CD4+ Helper T cells	Mediators of immune function secrete cytokines to enhance immune response	Glycolysis Acetyl CoA carboxylase (ACC)‐mediated *de novo* fatty acid (FA) synthesis
	CD8+ Cytotoxic T cells	Direct cytotoxic killing of cancer cells	Glycolysis Glutaminolysis
	Regulatory T cells	Suppress immune response	FAO
B cell	Resting/Activated	Secrete antibodies and perform humoral immunity	Glycolysis
DCs	Resting	Involved in antigen presentation and activation of T lymphocyte immune response	OXPHOS
	Activated		Glycolysis
NK cell		Regulate the adaptive immune response through the release of IFN‐γ in the early immune response	Glycolysis
Macrophages	M1 (classical activation)	Antigen presentation and pathogen clearance	Glycolysis Pentose phosphate pathway
	M2 (alternate activation)	Production of anti-inflammatory cytokines to promote immunosuppression and tumor progression	OXPHOS FAO
Neutrophils Mast cells	N1 (anti‐tumor) N2 (pro‐tumor)	Antitumor polarization induced by type 1 IFN TGF-β overexpressed by tumor cells polarizes neutrophils to a tumor-promoting phenotype Expression of MHC molecules, IgE Fc receptors, release of allergic mediators	Glycolysis FAO Glycolysis OXPHOS

### Dendritic cells

DCs have important functions that can bridge the innate and adaptive immune responses (154). DCs acquire full antigen-presenting cell (APC) function upon maturation, phagocytose and process non-self-antigens, and take a leading antitumor role in tumor immunity (155). Once mature, DCs up-regulate their antigen-presenting and co-stimulatory molecules, and when they receive distress or activation signals, they are activated and move to secondary lymphoid organs to start activating adaptive responses, such as transforming themselves into potent T-cell activators (156). Glycolysis serves as an essential role in promoting the activation of DCs, while activation of DCs also alters lipid metabolism and affects their function.

### Tumor-associated macrophages

In the early stage of tumorigenesis, macrophages from healthy tissues can inhibit the growth of tumor cells after activation. However, at the stage of tumor progression, macrophages recruited from the tumor microenvironment to the tumor area by chemokines and growth factors (157, 158), i.e., tumor-associated macrophages (TAMs), promote the growth, aggression and metastasis of tumor cells (159–161). Therefore, TAMs often represent markers of poor clinical prognosis and immunosuppression of tumors in the body (162, 163).TAMs have both classical (M1) and alternative (M2) subtypes, with M1 cells having immune-enhancing and cancer-suppressive effects and M2 the opposite (164). The above evidence implies that balancing M1 and M2 in TAMs is not negligible for immunotherapy.

### Natural killer (NK cells)

NK cells, as spectral killer cells, are the first line of defense of the organism. It does not require antigen pre-sensitization or antibody involvement to kill target cells in a straightforward, sensitive and fast response (165, 166). They have an important role in antitumor because of their ability to kill almost all common cancer cell types and multi-drug resistant tumor cells (167). They also control adaptive immunity by secreting cytokines, such as IFN-γ (168), affecting dendritic cells, macrophages and neutrophils (169). Interacting with a variety of other immune cells in the body, they regulate the body’s immune status and immune function.

### Tumor- associated neutrophils

Neutrophils regulate the inflammatory response by secreting cytokines and chemokines and are considered to be the first line of defense against different types of microorganisms. However, neutrophils can also cause harm to the body. Neutrophils can be recruited from the circulation into tumor tissue along chemokines generated by tumor cells and immune cells, among others, and are converted into TANs in TME (170). Similar to TAMs, TANs can be classified into N1 (antitumor) or N2 (tumor-promoting) phenotype (N2) and will shift between the two depending on TME characteristics (171, 172).TANs have been reported to have important effects on promoting tumor proliferation, invasion, angiogenesis, and metastasis (173–175), and therefore tan levels can be used as an indicator of poor patient prognosis (176). Neutrophils derive most of their energy from glycolysis, and enhanced glucose consumption has been shown to be critical for enhancing neutrophil survival and function (176, 177).

### Regulatory T cells (Treg cells)

CD4+ T cells and CD8+ T cells have a significant ability to suppress antitumor immune responses, recognizing and eliminating tumor cells by releasing suppressive cytokines, cytotoxic particles, and other mechanisms. Treg cells are a class of cell populations that control the autoimmune response and can form an immunosuppressive environment by secreting IL-10, TGF-β, and IL-35. These immunosuppressive properties coincide with the promotion of immune escape of tumor cells (178). Several experiments have verified that tumor patients usually have dysfunctional antitumor immune cells with a sharp rise in the amount of Treg cells (179–181). Interestingly, many tumor cells can exploit this suppressive mechanism and express multiple ligands (e.g. PD-L1, PD-L2) that help to absconds from T cells. Some articles indicate that Tregs are also heterogeneous, mainly: naturally occurring thymus-derived CD4+CD25+FOXP3+ Tregs (nTregs), and induced Treg cells (iTregs). There are three further subpopulations of iTregs: iTregs expressing FOXP3, Tr1 secreting CD4+FOXP3-IL-10, and Th3 expressing TGF-β (182, 183). iTregs exhibit completely different metabolic patterns depending on their activation status (184). iTregs, like M2 macrophages, depend mainly on fatty acid oxidation (FAO) of OXPHOS for energy (185).

## Metabolic reprogramming of immune cells

**Figure 3:**
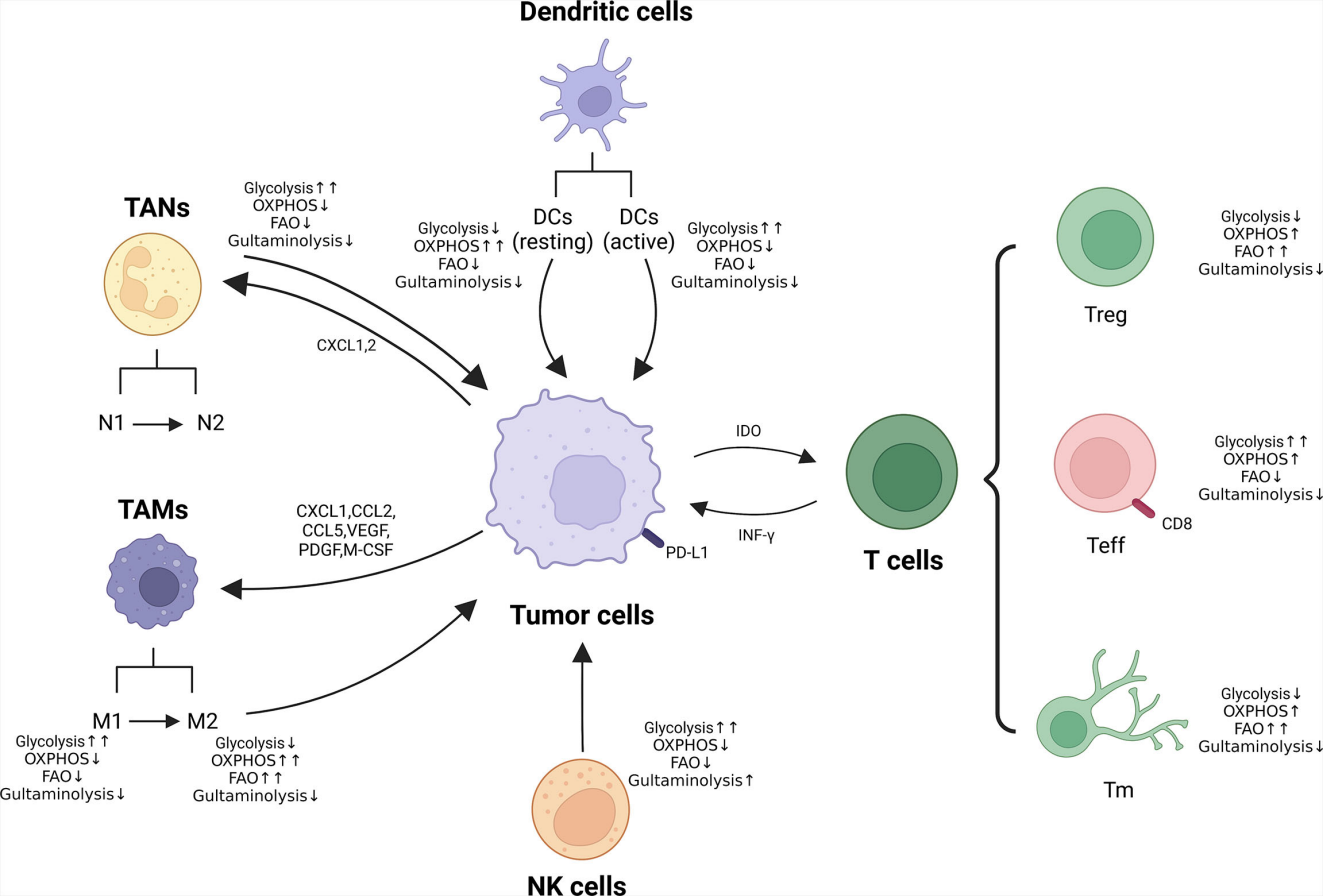
Metabolic reprogramming of immune cells in TME. Metabolic alterations of macrophages, neutrophils, NK cells and T cells (Treg, Teff and Tm) are presented. ↑↑: Significantly up-regulated; ↑: Up-regulated; ↓: Down-regulated.

Immunotherapy has brought a dramatic change in the treatment paradigm of tumors in the last decade, ensuring the reactivation of host defenses and playing an important strategic role in the fight against cancer. However, there are still problems of low efficiency and high adverse effects, the reason for which is incredibly significant is the metabolic reprogramming-mediated immunosuppression of the microenvironment (186, 187). It is well known that alterations in metabolism occur within cancer cells and that this metabolic disturbance not only affects their survival and proliferation signals, but also leads to a hyper acidic, nutrient-deficient and hypoxic TME (188, 189), which further exacerbates the metabolic reprogramming process of tumor cells and tumor ecotone immune cells. Recent studies have shown that immune cells also exhibit different metabolic patterns in various states of activation or stages of differentiation, which can regulate the phenotype, function and survival of immune cells (190–192). In addition, the environmental and metabolic state of the organism can also influence the phenotype and function of immune cells. Metabolic reprogramming mainly includes abnormalities in glucose metabolism, amino acid metabolism and lipid metabolism (193). Therefore, we will explore the impact of metabolic reprogramming of immune cells on carcinoma immunity in the carcinoma below (**Figure 3**).

### Glucose metabolism

Glucose metabolism is the principle productive mode of immune cells. Under aerobic conditions, glucose is transformed to pyruvate. It penetrates the tricarboxylic acid cycle (TCA) in the mitochondria, which then couples OXPHOS to produce large amounts of ATP, thereby providing the energy required for body metabolism (194). Under anaerobic or hypoxic conditions, glucose is transformed to pyruvate, which no longer penetrates the TCA, but synthesizes lactate in the cytoplasmic matrix to produce a small amount of ATP (195). The activation of immune cells is analogous to the Warburg effect in tumor cells, which also undergoes a metabolic reprogramming from a mitochondria-dominated oxidative phosphorylation process to aerobic glycolysis, with neutrophils, M1 macrophages, dendritic cells and other activated immune cells rapidly providing ATP and metabolic intermediates (196). The glycolytic intermediate glucose-6-phosphate also ameliorates the attunement of tumor cells to the stern microenvironment through the pentose phosphate pathway (PPP) and significantly affects the antitumor immune response of immune cells (197). Accurate information from the number of groups have shown that interference with OXPHOS, PPP of immune cells can suppress their immune function (198, 199).

It has been found that excessive glucose intake activates some helper T-cell Th17-related cytokines: TGF-β and RORγt, promoting exorbitant differentiation and stimulation of Th17 cells and triggering inflammatory responses *in vivo* (200). When lacking of glucose in the TME, the function of most immune cells becomes faulty. Inhibition of glycolytic capacity at depressed glucose levels activates the intracellular “energy receptor” AMPK kinase, which inhibits mTORC1 and HIF-1α, thus down-regulating the differentiation and function of DC, Teff and NK cells and promoting Treg differentiation and anti-inflammatory M2 macrophage formation (201–203). These analysis consequences intimate that glycolytic upregulation not only provides an intrinsic growth convenience for tumor cells, but also has an outward role in suppressing tumor immune surveillance.

From another perspective, the enhanced glycolysis of tumor cells is accompanied by the production of large amounts of metabolites such as lactic acid and CO2, which accumulate in the TME to form an acidic environment and further exert metabolic pressure on the infiltrating immune cells. The acidic environment: 1) impedes the extracellular transport of lactic acid in cytotoxic T lymphocytes (CTL) and NK cells, leading to intracellular acidification, which directly affects the proliferation and cytokine secretion of CTL and NK cells, leading to their lethality impaired (204, 205); 2) promotes the differentiation of initial T cells, MDSCs and TAMs toward the pro-tumor phenotype for differentiation and proliferation (205), which ultimately results in immune escape of tumors through multiple mechanisms. Recent clinical findings have shown that glycolytic activity in tumor cells is inversely correlated with host antitumor immune response and treatment outcome of immunotherapy (193). Patients with tumors that are difficult to control with peripatetic T-cell therapy have higher levels of aerobic glycolytic activity and have lower tissue TIL numbers and cytotoxic functions (206). Higher levels of lactate and concomitant acidified TME in tumors suppress immune cell function, abolish immune surveillance of the tumor, and ultimately result in immune escape.

### Amino acid metabolism

Glutamine (Gln) is the second most important intracellular nutrient after glucose, producing glutamate and ammonia under the action of glutaminase (GLS), which is subsequently converted to α-ketoglutarate catalyzed by GLS or transaminase and enters the mitochondria, where it undergoes OXPHOS through the TCA cycle and electron transport chain, producing Most of the ATP, and participate in nucleotide, amino acid and fatty acid synthesis (124). At the same time, Gln can be converted to glutathione, which is applied to maintain intracellular reactive oxygen species homeostasis and prevent its damage to biomolecules. Therefore, amino acid metabolism acts as an essential role in maintaining the growth and proliferation of tumors. Many tumor cells undergo reprogramming of amino acid metabolism, resulting in a deficiency of the corresponding amino acids in TME, resulting in impaired immune effector cell function. Targeting at tumor cell amino acid metabolism is one of the effective strategies to restore the immune response.

Activated T cells and macrophages also present enhanced Gln metabolism to maintain cell proliferation and immune response (207). Tumor cells have the capacity to prevent T cells from proliferation, activation and secretion of related cytokines through competitive consumption of glutamate, resulting in the formation of an immunosuppressive microenvironment. For example, in Gln-deficient microenvironment, renal cancer cells induce programmed death ligand-1, PD-L1 expression through activation of EGFR/ERK/c-Jun pathway, which inhibits IFN-γsecretion by T cells and allows tumor cells to evade immune killing (208). However, restriction of Gln during T cell activation leads to differentiation to CD8+ memory T cells (209), moreover, inhibition of GLS facilitated the differentiation and effector functions of Th1 and CTL but undermined the differentiation of Th17 cells (210). The mechanisms indicate the regulation of Gln metabolism in various cells in TME and how Gln affects T cell responses need to be further elucidated.

### Lipid metabolism

Lipid metabolism is a fundamental mode of energy supply in addition to glycolysis. It was found that increased fatty acid content in TME facilitates Treg production and that Treg relies on exogenous fatty acid uptake for immunosuppressive functions (211). Similarly, lipid accumulation in myeloid cells infiltrating TME induces a conversion to an immunosuppressive and anti-inflammatory phenotype through metabolic reprogramming, which may be partly derived from neighboring cancer cells with enhanced fatty acid synthesis. From another perspective, CD8+ T cells with higher levels of lipids in tumor patients up-regulated the expression of programmed death-1 (PD-1) receptor, which should generally show inhibitory effects; however, the combination of PD-1 inhibitors showed efficient antigen recognition and better antitumor results.

Fatty acid oxidation (FAO), is not the primary metabolic pathway (212). Nevertheless, the role of FAO in adjusting immune cell behavior cannot be overlooked; regulatory T cells (Treg cells), M2 macrophages, and memory T cells depend mainly on FAO-derived OXPHOS to produce energy. M2-like macrophages depend on FAO to meet the bioenergetic requirements to preserve their antitumor effects; inhibition of FAO promotes M1 polarization (213, 214). In addition, FAO serves as a significant section in the origination and preservation of memory T cells (Tm, memory T cells) (215). Investigators have verified that FAO is the metabolic energy foundation for the timely response of Tm to antigenic stimuli and facilitates the maintenance of normal Tm mitochondrial function and long-term cell survival. Importantly, FAO is also essential in upholding the equilibrium between Teff and Treg (216). FAO suppresses Teff cell activation, and up-regulates the expression of repressive PD1 receptor and CPT1A, which in turn attenuates IFN-γ secretion (217). Conversely, FAO genes such as CPT1A expression are up-regulated in Treg cells and FAO levels are elevated, providing energy to promote Treg cell production (216). FAO acts as a fundamental role in the regulation of innate and adaptive immune responses, which are largely determined by the diverse principal requirements of various immune cells. Consequently, figuring out the metabolism of distinct immune cells is crucial for a broad understanding of the mechanism of immune regulation of FAO.

It has also been indicated that inhibition of the cholesterol synthesis pathway in macrophages provokes the production of type I interferon responses, which initiate antiviral immune responses (218). ACAT1 is the major enzyme that catalyzes the synthesis of cholesteryl esters in CD8+ T cells, and pharmacological or genetic inhibition of ACAT1 increases intracellular cholesterol levels in melanoma tumor-infiltrating T lymphocytes (TIL), thereby inducing a superior immune response (219). The application of ACAT1, avasimibe (a small molecule inhibitor), in combination with a PD-1 inhibitor showed synergistic effects, significantly inhibiting the growth of tumor-bearing mice (219). However, recent studies have shown that high cholesterol levels in tumors can lead to T-cell dysfunction by activating endoplasmic reticulum stress (220). Therefore, despite the vital role of cholesterol for effector T-cell proliferation and metabolism, and although cholesterol acts as a crucial part in the proliferation and metabolism of effector T cells, targeting specific cholesterol metabolism requires further research.

## Crosstalk between CAFs and immune cells

**Figure 4:**
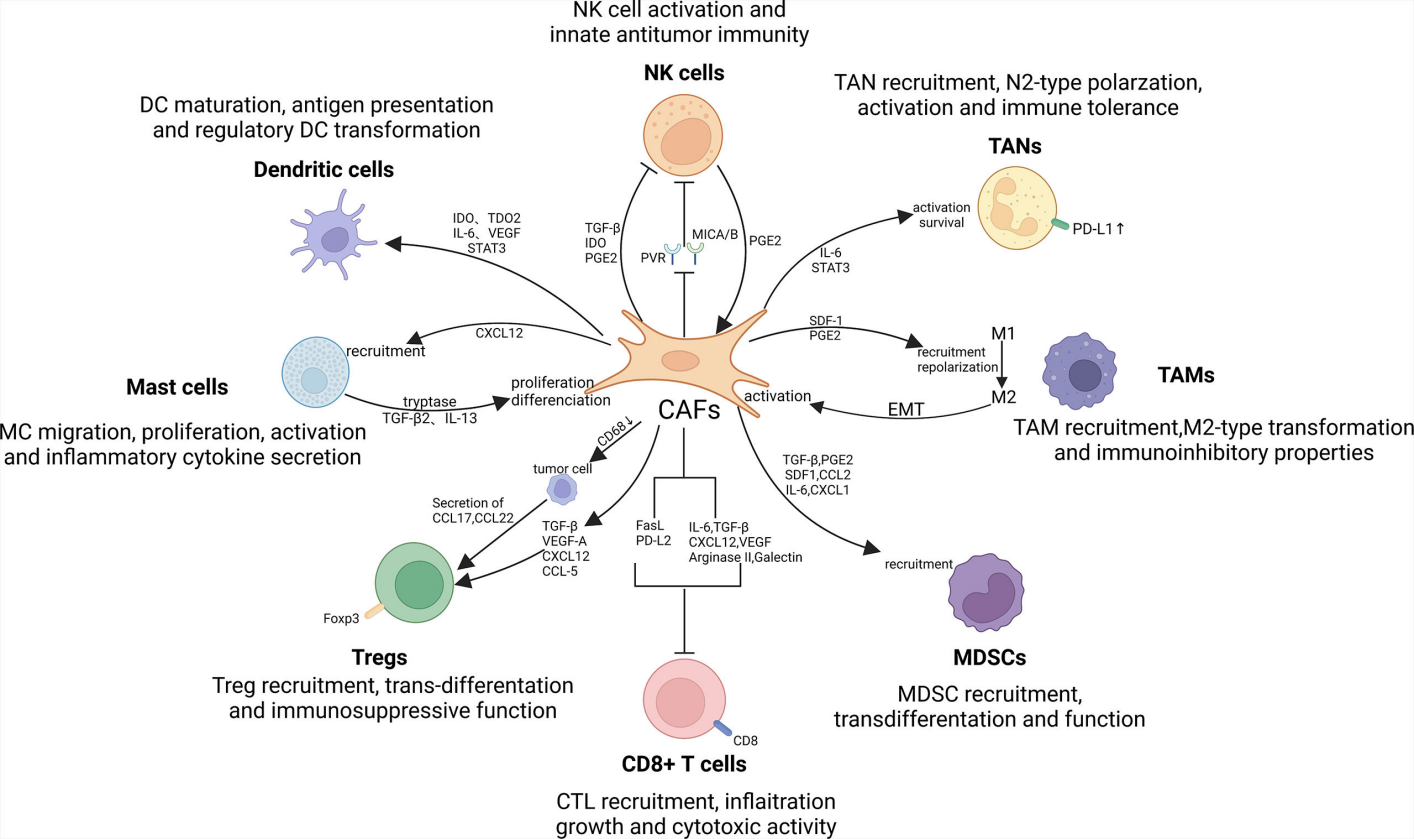
Crosstalk between CAFs and immune cells in the TIME. There exist significant interactions between CAFs and immune cells, such as tumor-associated macrophages (TAMs), tumor-associated neutrophils (TANs), mast cells (MCs), dendritic cells (DCs), myeloid-derived suppressor cells (MDSCs), natural killer (NK) cells and T lymphocytes. By secreting cytokines, chemokines, and other effector molecules, including TGF-β, CCL2, CXCL2, laminin, and MMP, CAFs can promote the involvement of immune cells in tumorigenesis and progression. Notably, TAMs, NK cells and MCs can in turn exert promoting effect on CAFs activation and function, thereby contributing to the formation of immune suppressive loops.

To date, accumulating evidence suggests that CAFs are critical for regulating the antitumor activity of immune cells in the TME, including innate and adaptive immune cells (221, 222). By secreting cytokines, chemokines, and other effector molecules, including TGF-β, CCL2, CXCL2, laminin, and MMP, CAFs can enhance the involvement of immune cells in tumorigenesis and progression (20, 223). In turn, some influences of several immune cells on CAF also attracted our attention (224, 225). In conclusion, just as abounding research has revealed that the interaction between CAFs and immune cells can modulate TIME, thereby suppressing antitumor immune responses (226, 227) (**Figure 4**).

### CAFs interacted with innate immune cells

#### Tumor-associated macrophages

The so-called TAMs have been divided into two manifest subsets called M1 and M2 (164). Type-1 TAM exert antitumor effects principally through the mediation of antibody-dependent cytotoxicity and secretion of ROS and TNF (228). However, in sharp contrast to the former, M2 macrophages mainly exhibit tumor-promoting activity. Studies have shown that this diametrically opposite effect is closely related to the promotion of tumor angiogenesis, immunosuppression, cancer cell aggression and spreading, and ECM reconstruction (164).

CAFs are mediated by a variety of regulatory molecules that encourage the TAM precursors and monocytes to recruit and simultaneously differentiation into type-M2 TAMs. Thus, CAFs can attenuate effector T cell responses and induce immunosuppression in the TME (229). Both Ksiazkiewicz M and Cohen N found that, in breast cancer, CAF is capable of promoting monocyte chemoattractant protein-1(MCP-1), SDF-1 and chitinase 3-like 1(Chi3L1) secretion. As a result, polarization of monocyte can be migrated and enhanced into M2 phenotype (230, 231). Several cytokines secreted by CAFs, including IL-8, IL-10, TGF-β, and CCL2, just as what have been revealed that, could enhance the monocyte recruitment and type-M2 TAM transformation (232–234).

As for the fact that CAFs can stimulate the immunosuppressive properties of TAMs, many studies have found that CAF-induced up-regulate the expression of PD-1 of type-M2 TAMs. Evidence suggests that high level expression of PD-1 in TAMs can reduce the phagocytosis to cancer cells and inhibit the infiltration and proliferation of T lymphocytes, implying that CAFs can induce restraint of not only innate but also adaptive antitumor immune responses to TAMs.

Likewise, TAMs with the M2 phenotype also have a counteracting effect on CAF, which can modulate CAF activation and progression (225, 235). Comito et al. (236) found that M2 macrophages can secrete IL-6 and SDF-1, and these factors enhance the advance of epithelial-mesenchymal transition and induce CAF activation. Activated CAFs can also further enhance TAM activity. Thus, a closed-loop pathway with both cancer-promoting and immunosuppressive effects quietly play its indispensable role in the TME. In addition, there is relevant evidence that TAM affects the differentiation and activation of MSCs, known as the precursors of CAF (237). TAMs can promote the transdifferentiation of MSCs into CAFs in terms of properties and functions and at the same time acquire a pro-inflammatory phenotype to reshape the inflammatory microenvironment (238).

#### Tumor−associated neutrophils

Similar to the classification of TAM, TAN can also be divided into antitumor phenotypes (N1) and tumor-promoting phenotypes (N2) (239–241). The main difference between N1 and N2 or the basis of classification lies in whether they can be activated by TGF-β and the different activation degrees (242). There is evidence that CXC chemokine receptor 2 (CXCR2) expressed by CAFs is a major force in mediating neutrophil recruitment to tumors. In short, this means that CAFs may promote the migration of TANs by relying on CXCR2 as a medium (243, 244). Cheng Y et al. found that the link between CAF and TAN also lies in the fact that the STAT3 signaling pathway in TAN can be activated by CAF-derived IL-6, which directly leads to PD-1/PD-L1 activation. When the expression is up-regulated, the activation of T lymphocytes can be inhibited and immune tolerance will soon be established (245). Unexpectedly, peripheral neutrophils are also regulated by CAF-secreted SDF-1α to migrate into tumor cells (245).

It should also be noticed that CAF-secreted cardiomyokine-like cytokine 1 (CLCF1) is crucial to promote the development of tumors. In advances in hepatocellular carcinoma research, polarization of TAN might be modulated by CAFs. The expression of CXCL-6 and TGF-β can be up-regulated by CLCF1, thereby inducing polarization of N2-type TAN, which contributes to tumor progression (246).

#### Mast cells

Taking prostate cancer as an example, CAFs can enhance MC proliferation and migration after estrogen overexpression and at the same time promote the secretion of inflammatory cytokines, which is a manifestation of tumor-promoting effect (247). Under the induction and catalysis of estrogen, the binding of CXCL12 to CXCR4 promotes the recruitment of MCs (247). In addition, Ma et al. (248) found their study stimulatory effects of MCs on CAFs. MC-secreted IL-13 and tryptase induce CAF proliferation in a manner distinct from TGF-β2-STAT6. Increased CAFs lead to the formation of fibrotic TMEs, with the direct consequence of suppressing antitumor immune and therapeutic responses (248). In addition, it has been reported that MCs in neurofibromas can enhance the proliferation and secretion of CAFs and promote CAF activity through the TGF-β pathway (249).

#### Natural killer cells

Emerging studies have indicated that CAFs induce direct or indirect restriction to NK cells *via* a variety of procedures, consisting of activating NK receptor, cytotoxic activity and cytokine secretion (31, 250).

*Via* PGE-2 and indoleamine 2,3-dioxygenase (IDO), NK cells are induced by CAF to switch to a quiescent phenotype and display a status characterized by unresponsiveness or even paralysis during antitumor immunity in hepatocellular carcinoma (251). NK cells are able to encourage the establishment of the immunosuppressive loop prompted by CAF through inducing PGE-2 discharge (252). By regulating the expression of NK cells’ energizing receptor-associated ligands, CAF could even restrain the activity and function of them. Whether the down-regulation of the MICA/B expression caused by CAF in melanoma on tumor cells (253), or CAF-mediated reduction of cell surface poliovirus receptor PVR expression, the most direct consequence is the inhibition of NK cell killing activity (254).

In the current discussion of the mechanism, TGF-β is widely recognized as the key linking CAFs to NK cells in TIME (255). Numerous investigations have demonstrated that TGF-β substantially restricts NK cell activation and cytotoxicity (256). Whether there are other related factors influence needs further research to explore and demonstrate.

#### Dendritic cells

Several studies have shown that CAFs of hepatocellular carcinoma are able to recruit natural DCs and stimulate their transdifferentiation into regulatory DCs (rDCs) through the activation of IL-6-STAT3 pathway. IDOs, for example, are incapacitated DCs that express low levels and present minoro antigen, but have the ability to secrete immunosuppressive effector cytokines (257). IDO is also critical to the proliferation of Treg cells, thereby suppressing T cell-mediated immune effects (258). In lung cancer, CAF releases IDO1 and tryptophan 2,3-dioxygenase (TDO2) under the induction of galectin-1. IDO1 and TDO2 are then degraded by tryptophan, which impairs the differentiation and normal immune function of DCs (259, 260). In addition, VEGF emerged by CAF participates in the anomalous differentiation of DCs and leads to damaged antigen presentation (261, 262).

### CAFs interacted with adaptive immune cells

#### T lymphocytes

Studies have shown that the level of Foxp3 expression on Treg cells is essential to restrain antitumor immune function (263). Several studies have found that CAF prompts Treg cells migration and significantly enhances the accumulation in the tumor cells (264). In regard to breast cancer, both the Karnoub AE team and the Tan W team found that the chemokine CCL5 determines the process of recruiting CD4+CD25+ Treg cells to CAFs (43, 265). Furthermore, down-regulation of CD68 expression in CAFs promoted tumor cell secretion of CCL17 and CCL22, two chemokines that directly increased Treg cell infiltration (266). It needs to be added that the growth factor released by CAFs, VEGF-A, is likely to implicate in the activation and preservation of Treg cells mediately or immediately (267, 268). Besides encouraging the migration and infiltration of Treg cells, the effects of CAFs on Treg cells also lie in inducing convention and exerting immunosuppressive effects as well. For example, CAF promotes the differentiation of naive T cells to relatively mature CD4 + CD25 + Treg cells through the induction of Foxp3 gene expression in T lymphocytes by secreting TGF-β (269).

Cytotoxic T lymphocytes (CTL) are considered to be the most critical components of antitumor immunity, also known as CD8 + T, and their well-known cytotoxic activity is mainly achieved by inducing tumor cell apoptosis (270, 271). Numerous studies have reported the crosstalk between CAFs and CTLs, especially the inhibitory effects of CAFs on CD8+ T cell infiltration, growth, and antitumor immunity (272). CAF activates pancreatic stellate cells (PSCs) through the secretion of cytokines such as CXCL12, which drives CD8+ T cells away from tumors, thereby reducing their infiltrating numbers in pancreatic tumors (273). When there is hypoxia in the microenvironment, CAFs can be stimulated to release angiogenic factors such as VEGF, resulting in a decrease in the expression of cell adhesion molecules on endothelial cells, making it difficult for circumferential CD8 + T cells to reach the tumor through the vasculature. Site exerts effects (274, 275). Hypoxic state or other physical barriers in the TME itself have also been shown to be closely related to the mediation of CAF (276). IL-6 and TGF-β secreted by CAFs can inhibit the recruitment of CD8+ T cells and counteract the cytotoxic activity of CTLs on tumor cells (277, 278). Arginase as well as galectin is important forces for CAF to restrict CD8 + T cells proliferation and destroy their immune function (279–281). Recent studies have pointed out that CAF promotes the reduction of the amount of CD8 + T cells and the improvement of tumor cell survival capability by inducing the expression of immune checkpoints in an indirect manner, thereby achieving the effect of weakening the antitumor response of effector T cells (229, 245, 282). Possible immune checkpoint molecules include factor associated suicide (FAS)/factor associated suicide ligand (FASL) and PD-1/programmed death-ligand 2 (PD-L2).

In conclusion, the effect of CAFs on T lymphocytes is relatively straightforward. It can promote the transition of naive T cells to a cancer-promoting phenotype, strengthen the function of immunosuppressive T lymphocytes, and inhibit the activity of effector T lymphocytes, thereby achieving immunosuppression. However, research on the effect of T lymphocytes on CAF is still uncharted territory, which may be a new direction for future research.

#### MDSCs

MDSCs, originating from the bone marrow, have been well known for their powerful immunosuppression of the TIME (283–285).

The infiltration and manufacturing of MDSC can be promoted by CAF through the discharge of diverse cytokines and chemokines. Thereby, antitumor activity of effector T lymphocytes is inhibited. Among them, CCL2 is a typical example, which can recruit MDSCs to migrate to tumor sites (282, 286, 287). The net result of increased M-MDSC aggregation is that the maturation of CD8+ T cell and the secretion of IFN-γ are restricted (288). IL-6 secreted by CAF instigates the differentiation of recruited monocytes into M-MDSC through STAT 3-dependent pathway, thereby inhibiting T cell proliferation and immune function (289). After interfering CAF with tranilast, Ohshio Y’s team found that the expressions of SDF-1, PGE2 and TGF-β1 secreted from CAF were significantly down-regulated, and the differentiation of primitive MDSCs showed a low-level state (290). In this way, SDF-1, PGE2 and TGF-β1 are highly likely to be involved in the differentiation of MDSC (290). Furthermore, CXCL1, secreted by CAFs, is also associated with the recruitment of PMN-MDSCs (291).

### CAFs in immune cell-mediated immune responses

CAFs play a significant role in the profound changes in ECM structure in tumors, and changes in ECM rigidity (or “compliance”) also have extraordinary impacts on tumor development and may regulate tumor infiltration and metastasis (119, 292–296). CAFs lead to ECM remodeling probably due to the progression of fibrosis, characterized by the degradation of type IV collagen accompanied by the deposition of type I and type III collagen (116, 118). One possible mechanism by which increased ECM rigidity promotes tumor infiltration and metastasis is the enhancement of growth factor-mediated cell migration (292, 293). This CAFs-modified ECM remodeling acts as a barrier, preventing immune cells from contacting tumor cells, thereby suppressing immune cell-mediated immune responses (297, 298). Therefore, CAFs-mediated ECM remodeling has been used as a predictor of clinical outcomes in patients in several clinical models. The progressive isolation of T cells from tumor cells during ECM remodeling has been demonstrated to be the primary immunosuppressive mechanism in various types of cancer (297, 299). In some types of tumors, higher T cell motility was observed in tumor areas where fibrosis had not progressed or was not fully fibrotic, whereas T-cell migration was poorer in areas of more intact fibrosis (300, 301). In addition, the order of the fibrous tissue surrounding the tumor epithelium determines the migration trajectory and migration speed of T cells to some extent, thus limiting their contact with tumor cells to exert immune effects (302). Therefore, we can infer that CAFs can restrict the movement of CD4+ and CD8+ T cells. In addition to T cells, CAFs-mediated ECM remodeling also affects other immune cell populations, such as regulation of macrophage polarization, and effects on MDSCs and DCs, but the specific mechanisms remain elusive (20). Follow-up studies should focus on the specific mechanisms of CAFs-mediated ECM remodeling in various immune cells to investigate the therapeutic targets for enhancing immune response in TME.

Apart from its barrier function that prevents immune cells from contacting with tumor cells, the dense ECM structure shaped by CAFs can also lead to changes in the efficient utilization of oxygen, thereby inducing a hypoxic state that affects nutrient uptake and induces changes in cellular metabolism, thereby influencing the TIME (303). Hypoxia is a recognized tumor immunomodulator. There is also evidence that hypoxia induces ECM deposition in hypoxic tumor regions, suggesting a positive feedback loop between hypoxia and ECM deposition (304, 305). CAFs may be involved in aberrant angiogenesis leading to a limited number of functional vessels, thus creating hypoxic zones and promoting immunosuppressive networks within the TME (303). Possible mechanisms by which CAFs regulate aberrant angiogenesis contain recruitment of tumor endothelial progenitor cells by secreting pro-angiogenic factors and by releasing SDF-1 in TME (306). Hypoxia can inhibit the infiltration of T cells in TIME, preventing them from making contact with tumor cells to mount an immune response, while also impairing the function of T cells (307). One mechanism of hypoxia-mediated T-cell suppression is the sustained activation of HIF-1α that negatively regulates T-cell receptor signaling, in part due to increased NF-κB activation (308). Multiple studies have demonstrated that hypoxia induced by CAFs interferes with T cell effector functions and allows tumor cells to escape from immune surveillance (309–312). Hypoxia also causes immunosuppression and contributes to immune tolerance *via* other immune cell populations such as TAMs and MDSCs (307). Possible mechanisms involved include selective upregulation of PD-L1 on MDSCs by hypoxia through HIF-1α binding to the HRE in the proximal promoter of PD-L1 (312).

In conclusion, CAFs play multiple roles in immune cell-mediated immune responses, but the specific mechanisms remain to be further investigated, which may provide new ideas for future drug research targeting CAFs.

## Immunotherapy strategies targeting CAFs

Considering the fact that CAFs exert their suppressive influences on tumor immunity utilizing multiple mechanisms and immune cell crosstalk, targeted therapies that target these cells are very promising. The quantity of preclinical trials to enhance or restore anti-cancer immune responses by targeting CAF therapy has augmented dramatically during recent decades. At present, CAFs-based immunotherapy mainly contains the following strategies: direct action on CAF targets to deplete CAFs, inhibition of the activation of CAFs and their functions. A brief summary of immunotherapy strategies for CAF in clinical and preclinical studies is given in the **Table 3**, and we will discuss part of them in the following section.

**Table 3:** Multiple clinical studies of CAF-targeted immunotherapy and related drugs.

Status	Cancer types	Drugs	Mechanisms	Biological effects	Combination therapy	Reference
Phase I	Brain glioblastoma multiforme	anti-TNC dsRNA (ATN-RNA)	Tenascin-C mRNA-targeted interference	Prolongs patients’ survival and restricts tumor recurrence	Surgery	20118657
Phase I	Pancreatic cancer	CCX872	CCL2-CCR2 signaling axis inhibition	Restricts immune suppression and improves clinical prognosis	FOLFIRINOX	317,318
Phase I	Breast, lung, HCC, CRC, pancreatic and renal cancer	NIS793 ABBV151	Blocking pan-TGF-β and GARP	Reverses tumor immunosuppression	Anti-PD-1 immunotherapy	23298232
Phase I	Recurrent epithelial ovarian cancer	Tocilizumab (monoclonal antibody)	IL-6-JAK/STAT3 signaling pathway inhibition	Enhances antitumor immunity and provides survival benefits	Carboplatin/Doxorubicin	26216383
Phase II	Colorectal cancer, Melanoma	Val-boroPro (talabostat)	FAP-targeted inhibitor small-molecules	Inhibit tumor growth and invasion, prolong patient survival	Cisplatin	19643020,18032930
Phase II	Pancreatic and hepatocellular cancer	Galunisertib	TGF-βR1 inhibition	Extends patient survival with minimal additional toxicity	Gemcitabine	30966391,30318515
Phase II	PDAC	Calcipotriol (vitamin D analog)	Vitamin D receptor activation and PSC deactivation	Reverses tumor immunosuppression	Anti-PD-1 immunotherapy	30778141
Phase II	Recurrent malignant glioma	^131^I-m81C6 (anti-tenascin mAb)	Radioimmunotherapy	Reverses tumor immunosuppression	NA	29443960
Phase II	Metastatic pancreatic cancer	Ruxolitinib (JAK inhibitor)	JAK-STA3 pathway inhibition	Inhibits tumor-promoting inflammation	Capecitabine	27053631
Phase III	PDAC	PEGPH20	Tumor stromal hyaluronan-targeted depletion	Prolongs patients’ survival with less systematic side effect	Gemcitabine and nabpaclitaxel	29235360

Multiple CAF-targeted immunotherapy strategies in different phase of clinical studies.

FOLFIRINOX (fluorouracil, leucovorin, irinotecan and oxaliplatin), TGF-βR1 transforming growth factor beta receptor 1, PD-L1 programmed death ligand 1, IL-6 interleukin-6, JAK Janus kinase, PDGFR platelet-derived growth factor receptor, CCX872 one of CCR2 antagonists, CCL2 C–C chemokine ligand 2, CCR2 C–C chemokine receptor 2, PEGPH20 a PEGylated human recombinant PH20 hyaluronidase, GARP glycoprotein A repetitions predominant protein, PDAC pancreatic ductal adenocarcinoma, HCC hepatocellular carcinoma, CRC colorectal cancer.

### Depletion of CAFs *via* cell surface markers

Current targeted CAF therapy focuses on the CAF-specific protein FAP (21, 29, 313). Recent studies have shown that ablation by FAP-expressing cells in a transgenic mouse model can lead to quick hypoxic necrosis and immunogenic tumor stromal cells of Lewis lung cancer, a process associated with the involvement of TNF-α and IFN-γ (314, 315). Previous studies have demonstrated that reducing or inhibiting FAP expression can reduce TME mesenchymal pro-connective tissue proliferation while enhancing the metabolic effects and cytotoxicity of CD8+ T cell-reliable killing of cancer cells. Several pioneer studies found that co-targeting CAF and cancer cells to increase T cell immunotherapy in a mouse-based model of lung cancer and malignant pleural mesothelioma patients with metastatic models showed unimpressive effect (316–319). Emerging research suggests that FAP is a crucial target for chimeric antigen receptor (CAR) therapy as well. CAR-T-cell therapy harnesses the host immune system to struggle against cancer. CAR-T-cell therapy counts on artificial receptors where immune cells are modified to express cancer-specific markers and injected into patients, in which they will specifically recognize and wipe out tumor cells (320, 321). Notably, however, FAP is expressed to varying degrees in cells in other tissues, such as pluripotent bone marrow stem cells, which can lead to serious side effects of CAR-T cell therapies. One study showed that relay transport of FAP-reactive T cells into mice suffering from various subcutaneous tumors exerted only finite antitumor consequences, but induced severe lethal osteotoxicity and cachexia in different strains of the mice (322, 323). Therefore, these fatal osteotoxicity and cachexia observed after immunotherapy with CAR-T cells targeting FAP emphasize its severe side effects as a universal target and should be used with caution in specific situations. However, as mentioned above (324), CAFs lack specific markers and therefore only a small number of treatments targeting CAFs have been applied in the clinic. In order to find out more concrete molecular targets for CAFs, we call for the need for more in-depth studies on the specific typing and heterogeneity of CAFs.

### CAFs activation to resting state transition

Given the difficulty of removing CAFs directly, converting CAFs in the activated state to the resting state is also a promising approach. In patients with pancreatic ductal adenocarcinoma (PDAC) and colon cancer, vitamin A deficiency led to PSC activation in PDAC patients (325–327), while a previous study showed that the vitamin D receptor (VDR) was a suppressor of PSC activation (325). And the activation of PSC led to the activation of CAFs. It has been demonstrated that supplementation with vitamin A or stimulation of VDR by drugs can inactivate PSCs (328). In PDAC patients, administration of the pleiotropic agent all-trans retinoic acid (ATRA) proved to inhibit the activation of PSCs, with the possible mechanism being the restoration of retinol levels in PSCs (327). In animal experiments, reversal of the phenotype of PSCs increased the infiltration of CD8+ T cells, thus enhancing the immune response (273). Thus, metabolic reprogramming of CAFs by vitamin A and VDR could effectively inhibit the activation of CAFs from exerting their corresponding effects in tumorigenesis and progression. Therefore, in certain types of tumor treatment, inactivation of PSC rather than direct elimination of CAFs may be a better therapeutic modality.

### Immunotherapy in combination with CAF derivatives

Given the fact that crosstalk between CAFs and other immune cells serves as a crucial role in the induction of immunosuppression in TME, it seems more feasible to inhibit the activation of CAFs and limit the crosstalk between CAFs and immune cells by targeting key effector molecules associated with CAFs, for instance, growth factors, signaling pathways and cytokines (324). Immunotherapy is sometimes ineffective in some specific cancers, such as pancreatic ductal carcinoma, so researchers have tried combining CAF-derived cytokines or chemokines with immunotherapy in an attempt to enhance the efficacy of immunotherapy (20). The researchers found that after administration of AMD3100 for inhibition of chemokine (C-X-C motif) ligand 12 (CXCL12) receptor 4, T cells were rapidly recruited in cancer cells and acted synergistically with α-PD-L1 to dramatically reduce cancer cell proliferation and metastasis. This combination of immunotherapy with CAF-derived cytokines or chemokines somewhat avoids the immune evasion caused by the crosstalk between CAFs and immune cells (329, 330). Previous studies have demonstrated that TGF-β acts as a critical role in the activation of CAFs and the crosstalk between CAFs and immune cells, suggesting that inhibition of TGF-β may be able to restore the impaired immune response to TME (331, 332). The clinical importance of immunotherapy is underscored by the fact that clinical and preclinical studies of multiple TGF-β-associated immunotherapies are currently underway. Previous studies have found that in mice using specific models, combined treatment with blockade of TGF-β and anti-PD-L1 antibodies restrained signaling of TGF-β in stromal cells, promoting T cell recruitment to tumor centers and subsequently provoking a robust antitumor immune response (224, 255). Similarly, in several phase I clinical trials, it was noticed that high-dose tocilizumab assists in stimulating the activation of CD8+ T cell and raising the expression of antitumor-connected effectors (e.g. IFN-γ and TNF-α), thus strengthening antitumor immunity (333). Different patients require specific treatment strategies, so gene identification is critical. For patients with lung squamous cell carcinoma (LUSC), E2 factor-related gene signatures can help screen out high-risk patients so that they can be assigned personalized treatment strategies (334).

Some other possible therapeutic strategies include limiting CAF- incurred ECM reshaping in TME. A typical example is retinoic acid (RA), a small molecule derivative of vitamin A, which affects the immunosuppressive properties of CAFs by inhibiting their IL-6 and ECM production (335). Despite the remarkable progress made so far, more research is required to discover other possible therapeutics targeting CAFs and their crosstalk network with immune cells, which may lead to new ideas for future antitumor immunotherapy and be a boon for cancer patients.

## Conclusions

Tumorigenesis and progression require metabolic reprogramming of TME. Furthermore, solid tumors are usually considered as metabolically heterogeneous diseases, in which metabolic reprogramming occurs in CAFs and immune cells, together with crosstalk between them ensure continuous cancer cell growth and proliferation. In this biological context, CAFs and immune cells may stand for the major cell types regulating endosmosis and interactions in cancer tissues. Currently, the prevalent metabolic circuit plasticity is considered to be the most vital limiting factor for successful metabolic inhibition, rendering single-targeted metabolic pathways ineffective. Multiple metabolic inhibition is a promising strategy to overcome this problem, and although reliable preclinical data are supporting (336), clinical efficacy assessment of multiple metabolic inhibitor strategies is still in its infancy. CAFs affect immune cell activity in numerous ways, and metabolic reprogramming in TME leads to immune effector cell dysfunction. These interactions further reinforce the immunosuppressive effects in TME, leading to rampant proliferation and metastasis of tumor cells. Future research directions may need to explore multiple metabolic inhibition in CAFs and immune cells as well as strategies related to immunotherapy. In particular, the maturing of strategies dedicated to inhibiting metabolic reprogramming of CAFs and immune cells and reducing metabolic crosstalk between them could help to eliminate proliferation in the cancer network and enhance immune cell-mediated immune responses.

## Author contributions

YZ: Literature retrieval, writing the draft manuscript, and literature statistics and tabulation. LW: Writing, revising the manuscript, and designing the figures. XL: Writing, revising the manuscript, and designing the figures. XH: Designing the figures. JY: Concept of the study. All authors contributed to the article and approved the submitted version.

## Funding

This work was supported by Social Development Science and Technology Special Project of Kunshan (KS1935).

## Conflict of interest

The authors declare that the research was conducted in the absence of any commercial or financial relationships that could be construed as a potential conflict of interest.

## Publisher’s note

All claims expressed in this article are solely those of the authors and do not necessarily represent those of their affiliated organizations, or those of the publisher, the editors and the reviewers. Any product that may be evaluated in this article, or claim that may be made by its manufacturer, is not guaranteed or endorsed by the publisher.

## Glossary

**Table:**

TME	tumor microenvironment
ECM	extracellular matrix
CAFs	cancer-associated fibroblasts
TCA	tricarboxylic acid cycle
LDH/LDHA	lactate dehydrogenase
HSCs	hepatic stellate cells
PSCs	pancreatic stellate cells
TA-MSCs	tumor-associated mesenchymal stem cells
FAP	fibroblast activation protein
FSP1/S100A4	fibroblast-specific proteins 1;
rCAFs	cancer-restraining cancer-associated fibroblasts
scRNA-seq	singlecell RNA sequencing
OXPHOS	oxidative phosphorylation
GLUT-1	glucose transporter 1
PK	pyruvate kinase
PKM2	pyruvate kinase M2
IDH3a	isocitrate dehydrogenase 3
Cav-1	Caveolin-1
TFAM	transcription factor A;
TNBC	triple-negative breast cancer
OSCC	oral squamous carcinoma
SDH	succinate dehydrogenase
FH	fumarate hydratase
Gln	Glutamine
GS	glutamine synthetase
AKT2	protein kinase B
FAs	fatty acids
CRC	colorectal cancer
FASN	Fatty acid synthase
TIME	tumor immune microenvironment
APC	antigen presenting cell
TAMs	tumor associated macrophages
FAO	fatty acid oxidation
CTL	cytotoxic T lymphocytes
GLS	glutaminase
PD-1	programmed death-1
Tm	memory T cells
TIL	tumorinfiltrating T lymphocytes
MCP-1	monocyte chemoattractant protein-1;
Chi3L 1	chitinase 3-like 1
CXCR2	CXC chemokine receptor 2
CLCF1	cardiomyokine-like cytokine 1
IDO	indoleamine 2, 3-dioxygenase
rDCs	regulatory DCs
TDO2	tryptophan 2, 3-dioxygenase
CTL	Cytotoxic T lymphocytes
PSCs	pancreatic stellate cells
FAS	factor associated suicide;
FASL	factor associated suicide ligand
PD-L2	PD-1/programmed death ligand 2
CAR	chimeric antigen receptor
PDAC	pancreatic ductal adenocarcinoma
VDR	vitamin D receptor
ATRA	all-trans retinoic acid;
LUSC	lung squamous cell carcinoma
RA	retinoic acid.
